# Integrating MRI Volume and Plasma *p* ‐Tau217 for Amyloid Risk Stratification in Early Stage Alzheimer’s Disease

**DOI:** 10.1002/alz70861_108109

**Published:** 2025-12-23

**Authors:** Sohyun YIM, Kichang Kwak, Sang Won Seo

**Affiliations:** ^1^ Samsung Medical Center, Gangnam‐gu, Seoul Korea, Republic of (South); ^2^ BeauBrain Healthcare Inc, Seoul, Seoul Korea, Republic of (South); ^3^ SAIHST, Sungkyunkwan University, Seoul Korea, Republic of (South); ^4^ Samsung Medical Center, Sungkyunkwan University School of Medicine, Seoul Korea, Republic of (South); ^5^ Alzheimer’s Disease Convergence Research Center, Samsung Medical Center, Seoul Korea, Republic of (South)

## Abstract

**Background:**

Accurately detecting amyloid‐β (Aβ) accumulation is critical for identifying patients with early stage Alzheimer’s disease (AD) who may benefit from Aβ‐targeted therapies. While Aβ positron emission tomography (PET) and cerebrospinal fluid (CSF) biomarkers are reliable, their high cost and invasiveness limit widespread use. This study evaluated a two‐stage diagnostic workflow integrating magnetic resonance imaging (MRI)‐based brain atrophy assessments and plasma *p* ‐Tau217 testing for predicting Aβ PET positivity in patients with early‐stage AD.

**Method:**

This prospective cohort study was conducted in a tertiary hospital setting, including participants diagnosed with mild cognitive impairment (MCI) or early Alzheimer‐type dementia (ATD) from the K‐ROAD cohort and ADNI cohort. A random forest classifier was developed to predict Aβ PET positivity using MRI‐derived brain atrophy patterns and *APOE* ε4 status. Participants were stratified into low‐, intermediate‐, and high‐risk groups for Aβ PET positivity based on the prediction from this classifier. Additional plasma *p* ‐Tau217 testing was then applied exclusively to individuals in the intermediate‐risk group. Diagnostic performance was assessed using positive predictive value (PPV), and negative predictive value (NPV).

**Result:**

The study included 807 participants form K‐ROAD (median age: 72.0 years, 58.7% female) and 230 from ADNI (median age: 70.9 years, 49.1% female). In the first stage, using the 95% sensitivity/specificity strategy, the NPV in the low‐risk group was 94.7% (91.7–97.7%) in K‐ROAD and 99.0% (97.0‐100.0%) in ADNI. The PPV in the high‐risk group was 97.6% (95.9‐99.3%) in K‐ROAD and 98.8% (96.5‐100.0%) in ADNI. The intermediate‐risk group comprised 33.3% in K‐ROAD and 20.9% in ADNI. In the second stage, plasma *p* ‐Tau217 testing for intermediate‐risk group achieved a PPV of 92.5% (88.7‐96.3%) and an NPV of 83.1% (75.0‐91.2%) in K‐ROAD. In ADNI, the PPV was 90.0% (79.0‐100.0%) and the NPV was 83.3% (66.1‐100.0%). The overall accuracy of the workflow was 94.2% (92.6‐95.8%) in K‐ROAD and 96.5% (94.1‐98.9) in ADNI.

**Conclusion:**

This study demonstrates that the two‐stage diagnostic workflow improves diagnostic accuracy for predicting Aβ PET positivity in early‐stage AD. Despite some limitations, this approach provides a cost‐effective and scalable strategy for early detection and optimized healthcare resource allocation.

